# Uncovering the deubiquitinase activity landscape of breast cancer

**DOI:** 10.18632/oncoscience.518

**Published:** 2020-11-09

**Authors:** Sijia Liu, Peter ten Dijke

**Affiliations:** ^1^Department of Cell and Chemical Biology, Leiden University Medical Center, Leiden, Netherlands; ^2^Oncode Institute, Leiden University Medical Center, Leiden, Netherlands

**Keywords:** DUB, activity-based probe, breast cancer, UCHL1, TGFβ

## Abstract

Breast cancer is a highly heterogeneous disease with dynamic changes in the tumor microenvironment. Precision medicine will in the future provide the possibility to treat each individual cancer patient with the right (combination) therapy specifically tailored to personal needs. However, in order to accomplish this, more accurate biomarkers for precise diagnosis, prognosis, therapy response, and target-specific drugs are required. Although an increasing number of (epi)genetic driving alterations have been reported in breast cancer, the major stumbling block for clinical application of many of them is that they are difficult to therapeutically target. Deubiquitinases (DUBs) are emerging drug targets that play important roles in cancer progression. Hence, we devoted our efforts to uncover the global DUB activity landscape of breast cancer in order to discover potential novel biomarkers or therapeutic targets. We developed a specific DUB activity-based inhibitor and probe and applied it to obtain new insights into breast cancer.

Breast cancer is still the most prevalent cancer among woman worldwide, approximately one in eight women will develop breast cancer during their life [1]. As a highly heterogeneous disease, breast cancer has been classified into five subtypes based on the histological and molecular characteristics: triple-negative, human epidermal growth factor receptor 2 (HER2)-enriched, luminal B-like HER2 positive, luminal B-like HER2 negative, and luminal A-like [2-3]. Among all the subtypes, triple negative breast cancer (TNBC), which lacks estrogen receptor (ER), progesterone receptor (PR) and HER2 expression, are the most metastatic and challenging subtype of breast cancer to treat. Metastasis is the main cause of death in breast cancer with little to no curable therapeutic options until now. Although there are some treatments that can prolong survival and control treatment-associated toxicity, almost all the patients with metastatic breast cancer will die in 2-3 years [3]. Therefore, more clinical meaningful molecular targets and specific low-toxicity drugs are urgently needed for the treatment of advanced breast cancer patients.

DUBs are a large group of proteases that play important role in ubiquitin system by removing the covalent attachment of a 8.5 kDa protein, called ubiquitin from proteins and other molecules. Ubiquitin conjugation can regulate the stability, activities and/or subcellular localization of substrates [4]. More than 100 human DUBs have been identified and classified into seven evolutionarily conserved families, over 90% of them are cysteine proteases containing the catalytic triad [5]. The well-defined active sites and catalytic common feature render DUBs to become attractive targets to develop activity-based probes (ABPs) and inhibitors [6]. The fluorogenic ABPs enable quick clinical samples profiling and they can be used in high-throughput drug screens [7]. The biotinylated ABPs allow accurate quantitative profiling of precious clinical samples when they are combined with mass-spectrometry (MS)-based proteomics [8].

Using these powerful tools, we established two platforms to perform DUB activity screens in 52 breast cancer cell lines that encompassed all the subtypes, and 52 breast cancer patients tissues that included ER positive and negative subtypes [9]. In one platform we used 5-carboxytetramethylrhodamine (TAMRA)-ubiquitin-vinyl methyl ester (VME) ABP, which is labeled on the N-terminus with a TAMRA fluorescent dye and equipped with a reactive carboxy (C)-terminal VME warhead (Figure 1A). In the other parallel DUB activity profiling we employed Biotin-ubiquitin-VME ABP combined with liquid chromatography (LC)/MS-MS analysis (Figure 1B). TAMRA ABP is like a Polaroid which can take a snapshot of global DUBs activity landscape, while Biotin ABP is like a microscope that can provide high-resolution photos of each DUB.

Based on two DUB activity screens, we identified ubiquitin carboxy-terminal hydrolase L1 (UCHL1) as a highly active DUB in TNBC cell lines and biopsies from ER negative breast cancer patients. In subsequent studies using mice and zebrafish xenograft breast cancer invasion/metastasis models, we were able to ascribe a metastasis-promoting function to UCHL1. Mechanistically, we found UCHL1 could promote the transforming growth factor-β (TGFβ)-SMAD canonical signaling pathway by protecting the cell surface TGFβ type I receptor and its downstream intracellular effector SMAD2 from proteasomal degradation. We further synthesized a specific small molecule UCHL1 activity inhibitor and demonstrated its powerful inhibitory effect on TGFβ/SMAD-induced signaling responses and TNBC metastasis. Besides, we detected significantly higher UCHL1 level in the serum of TNBC patients than healthy donor samples. UCHL1 was detected in vesicles secreted by cells called exosomes. We are now working to develop a specific UCHL1 activity probe that can be used to obtain more mechanistic insights into UCHL1 function and as biomarker. Our overarching aim is to translate our fundamental discovery into clinical application to improve the life quality of breast cancer patients.

**Figure 1:**
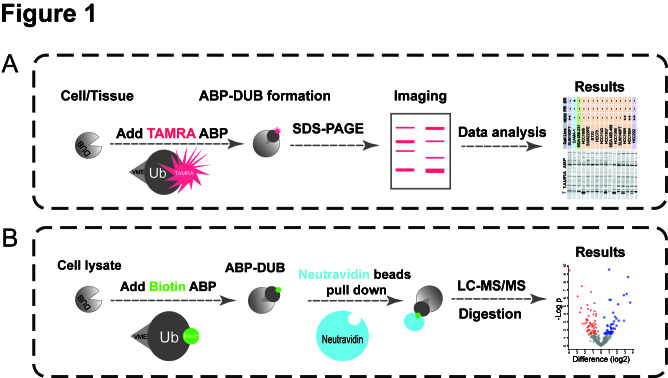
Schematic overview of DUB activity profiling with ABPs in breast cancer.
